# Hyaluronan Synthase: The Mechanism of Initiation at the Reducing End and a Pendulum Model for Polysaccharide Translocation to the Cell Exterior

**DOI:** 10.1155/2015/367579

**Published:** 2015-09-10

**Authors:** Paul H. Weigel

**Affiliations:** Department of Biochemistry & Molecular Biology, The Oklahoma Center for Medical Glycobiology, University of Oklahoma Health Sciences Center, Oklahoma City, OK 73190, USA

## Abstract

Hyaluronan (HA) biosynthesis has been studied for over six decades, but our understanding of the biochemical details of how HA synthase (HAS) assembles HA is still incomplete. Class I family members include mammalian and streptococcal HASs, the focus of this review, which add new intracellular sugar-UDPs at the reducing end of growing hyaluronyl-UDP chains. HA-producing cells typically create extracellular HA coats (capsules) and also secrete HA into the surrounding space. Since HAS contains multiple transmembrane domains and is lipid-dependent, we proposed in 1999 that it creates an intraprotein HAS-lipid pore through which a growing HA-UDP chain is translocated continuously across the cell membrane to the exterior. We review here the evidence for a synthase pore-mediated polysaccharide translocation process and describe a possible mechanism (the Pendulum Model) and potential energy sources to drive this ATP-independent process. HA synthases also synthesize chitin oligosaccharides, which are created by cleavage of novel oligo-chitosyl-UDP products. The synthesis of chitin-UDP oligomers by HAS confirms the reducing end mechanism for sugar addition during HA assembly by streptococcal and mammalian Class I enzymes. These new findings indicate the possibility that HA biosynthesis is initiated by the ability of HAS to use chitin-UDP oligomers as self-primers.

## 1. Introduction and Overview of HA Biosynthesis

Cell-free biosynthesis of HA was demonstrated in 1959 using* Streptococcus* membranes [1]. The enzyme responsible, HA synthase (HAS), is a membrane protein that requires only Mg^+2^ and two sugar-UDP substrates (GlcUA-UDP and GlcNAc-UDP) to polymerize HA chains. (To be consistent in using the standard convention of showing the reducing end of any glycan or saccharide to the right, we do not use the normal convention for nucleotide-sugars (e.g., UDP-GlcNAc); instead HA-UDP, GlcNAc-UDP, and GlcUA-UDP are abbreviated to show their reducing ends to the right.) No one was able to identify any streptococcal or eukaryotic HA synthase gene until 1993 when the* hasA* gene was identified and cloned, and the* S. pyogenes* HAS protein was expressed [2–4]. Identification of the* hasA* gene and the biochemical demonstration that only the HAS protein was required to synthesize HA [5] then led to the identification of* hasA* genes in* S. equisimilis* [6] and* S. uberis *[7] and vertebrate homologues of these HAS genes in many species [8–10]. The first active HAS was purified when the recombinant enzymes from Group A (SpHAS) and Group C (SeHAS)* Streptococcus* were overexpressed in* E. coli* SURE cells [11].

Mammalian genomes have three different HAS genes (HAS1, HAS2, and HAS3) that are expressed at specific times and specific tissues during development, aging, wound healing, and under normal or pathologic conditions or in diseases such as cancer [12, 13]. HA, which is found in only some prokaryotes but is a general ubiquitous extracellular matrix component in vertebrates [14, 15], is a linear heteropolysaccharide composed of the repeating disaccharide: (-3)-*β*-D-*N*-acetylglucosamine-*β*(1,4)-D-glucuronic acid-*β*(1-). This unsulfated glycosaminoglycan is a major component in cartilage and dermis, and in synovial and vitreous fluids. HA plays an important role during fertilization, embryogenesis, development, and differentiation [16, 17] and is also involved in many diverse cellular functions and behaviors, such as cell migration, phagocytosis, and proteoglycan assembly [18]. Additionally, HA plays important roles during wound healing and is used as a drug delivery vehicle, a cosmetic ingredient, and an analgesic device [19–21].

All known HASs, except one, are related structurally, show high sequence identity or similarity, share similar multi-membrane-domain organizations (with 6–8 membrane domains), predicted topologies, and processive mechanisms, and constitute a large family, the Class I HASs [10, 22]. The only known Class II HAS, from* Pasteurella multocida*, is different from Class I HASs in membrane attachment (having a single membrane domain), gene and protein sequence, domain organization, and having a distributive (nonprocessive) mechanism. This review focuses on the characteristics of Class I streptococcal and mammalian HASs.

**Figure 1:**
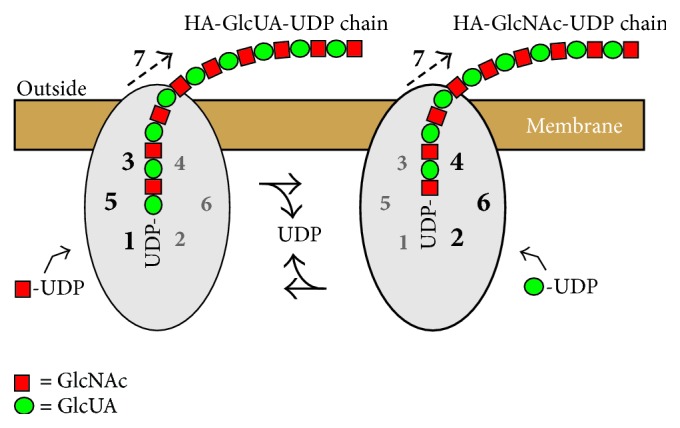
Schematic model of HAS showing the functions needed for HA chain growth at the reducing end and transfer to the cell surface. HAS uses multiple discrete functions (numbers 1–7) to assemble each HA disaccharide (red squares are GlcNAc and green circles are GlcUA). The same HAS protein is indicated in two different situations, at sequential times, as it alternately adds HA-GlcUA-UDP to a new GlcNAc-UDP, using functions 1, 3, and 5 (left), and then adds HA-GlcNAc-UDP to a new GlcUA-UDP, using functions 2, 4, and 6 (right). In this example (variant 1; Table 1) the sugar-UDPs are sequentially added in a continuous alternating manner and each cohort of needed functions cycles between being active (larger black numbers) and inactive (smaller gray numbers) within the active site domains (gray ovals). The functions required to add GlcNAc-UDP to HA-GlcUA-UDP are (left): 1, GlcNAc-UDP acceptor binding; 3, HA-GlcUA-UDP donor binding; 5, HA-GlcUA-UDP: GlcNAc-UDP, *β*1,3(HA-GlcUA-) transferase; and 7, HA translocation through the membrane. The functions required to add GlcUA-UDP to HA-GlcNAc-UDP are (right): 2, GlcUA-UDP acceptor binding; 4, HA-GlcNAc-UDP donor binding; 6, HA-GlcNAc-UDP: GlcUA-UDP, *β*1,4(HA-GlcNAc-) transferase; and 7, HA translocation.

Streptococcal and mammalian HASs in membranes [23–26] or as purified enzymes [27] elongate HA at the reducing end and do not require an exogenous primer to begin HA synthesis. HAS initiates biosynthesis using just the two sugar-UDP substrates, although we now know that the enzyme makes a self-primer using only GlcNAc-UDP (described below). HAS is an unusual enzyme in that it uses four substrates (i.e., two sugar-nucleotides and two types of HA-UDP chains, with either GlcUA or GlcNAc at the reducing end) and two glycosyltransferase activities within the same protein. DNA and RNA polymerases utilize template molecules to direct synthesis of products with only one type of bond between monomers. Heteropolysaccharide synthases, such as HAS (which makes a [GlcNAc(*β*1,4)GlcUA(*β*1,3)]_*n*_-UDP polymer), create* de novo* two different glycoside linkages in an alternating manner. The HA product after each sugar addition then becomes a substrate for the next sugar addition. In the presence of exogenous precursors, membrane-bound HASs use at least seven binding or catalytic functions (Figure 1) to synthesize disaccharide units at the reducing end of a growing HA-UDP chain. Class I HAS enzymes are processive; they do not rebind and extend HA chains once they are released.

**Figure 2:**
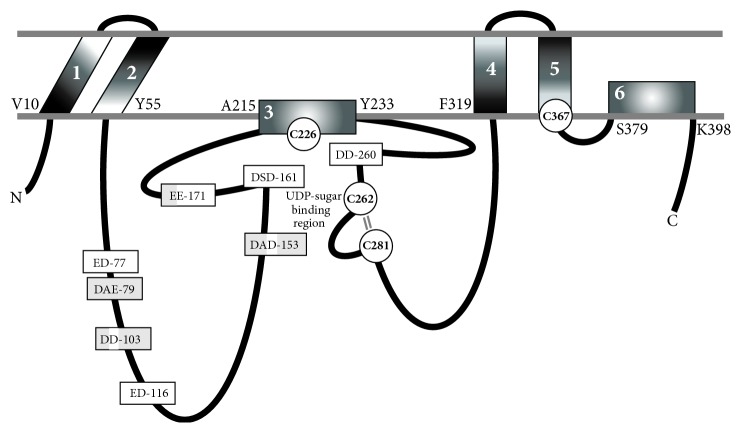
Membrane organization of HAS domains and conserved potential glycosyl-UDP binding regions. The experimentally determined topology of SpHAS [28] is modified to incorporate the discovery [46] that all four Cys residues of SeHAS (white circles) are at the membrane-protein interface and are located in or very near to the sugar-UDP binding sites. These four Cys residues are positionally conserved in the Class I HAS family. The SeHAS numbering shows the amino acids at the cytoplasmic junctions of the six MDs (white numbers 1–6). The parallel lines (gray) between C262 and C281 indicate the close proximity (~5 A) of these residues; they are not disulfide bonded. Eight “DXD”- or “XDD”-equivalent motifs in SeHAS, potential glycosyl-UDP binding sites (rectangle boxes), are either conserved just among the streptococcal enzymes (light gray) or also among the eukaryotic HASs (white); a few exceptions are discussed in the text. In some motifs, a streptococcal acidic residue is shaded white to indicate its conservation in the HAS family.

SpHAS, the only Class I HAS whose topology has been determined experimentally [28], has the N- and C-terminus and majority of the SpHAS protein inside the cell (Figure 2). StrepHASs have six membrane domains (MDs), four of which pass through the membrane giving two small loops of the protein exposed to the extracellular side. The other two MDs, one within the large central catalytic domain and one in the C-terminal one-third of the protein, interact with the membrane as amphipathic helices or reentrant loops but do not appear to span the membrane. The presence of two MDs in HAS that are amphipathic and do not cross the membrane is intriguing because these might be particularly well suited for the formation of an intraprotein pore. Vertebrate HASs contain an additional C-terminal region of ~130–160 aa with two trans-MDs. The amino ~75% of the larger eukaryotic HAS family members is homologous to SpHAS with the same predicted domain organization, so the overall topological organization of all the Class I HASs is predicted to be similar [10].

## 2. HAS Activity Is Regulated by Its Lipid Environment

Radiation inactivation studies [29] showed that the “active unit” mass of SeHAS or SpHAS is ~23 kDa more than a monomer, but smaller than a HAS dimer. The additional 23 kDa was identified as phospholipid (CL). The active streptococcal enzymes are HAS protein monomers in complex with 14–18 molecules of CL or other phospholipids. Similarly, active XlHAS1 is a monomer of ~69 kDa with an additional ~20 kDa of unknown components [30], probably phospholipid although this was not confirmed. Kinetic characterization of purified StrepHASs [31] and human HAS2 [32] confirmed that activity of these enzymes is regulated by, or dependent on, lipids. Purified StrepHASs have low activity without lipid and are activated ~10-fold by exogenous CL [11, 33], with high specificity for particular fatty acyl chains [34]. SeHAS is highly activated by oleoyl (C18:1) CL, but almost completely inactive with myristyl (C14:0) CL. The activity of purified human HAS2 in reconstituted liposomes is greatly influenced by cholesterol and the available lipids [32] and manipulating plasma membrane cholesterol content in different cell types causes them to make less HA [35, 36]. Thus, Class I HASs are either lipid-dependent or regulated by their lipid and cholesterol microenvironment. Strong positive modulation by cholesterol might also serve to minimize intracellular HA synthesis, which could be detrimental to many cellular pathways and functions if in excess.

## 3. Mammalian HAS Activity Is Regulated by Precursor Availability, Posttranslational Modifications and Protein-Protein Interactions

In streptococcal [37],* B. subtilis* [38], or mammalian [39] cells expressing HAS and making HA, the consumption of the two precursor sugar-UDPs is extraordinarily high compared to cells not making HA. To enable HA synthesis cells must have greater expression levels of the biosynthetic enzymes and greater flux rates in the precursor metabolic pathways; this often results in higher steady-state precursor concentrations, but a more important factor is that the rate of precursor synthesis supports the high rate of HAS precursor use. HAS regulation in mammalian cells is more complicated than in bacteria and several groups have identified a range of different mechanisms, including transcriptional and posttranslational control [40, 41]. Mammalian HAS2 has been studied the most and is regulated posttranslationally by phosphorylation [42], O-GlcNAcylation, and GlcNAc-UDP levels [43, 44], and by ubiquitination and dimerization [45]. It is not known if any of these regulatory mechanisms alters HAS monosaccharide assembly activity or HA translocation activity, but since these two functions are coupled, altering one activity is expected to alter both.

## 4. HA Synthases Elongate HA at the Reducing End

Stoolmiller and Dorfman [47] reported that the SpHAS adds new sugars to the nonreducing end, but other studies with membranes from streptococci [24] or eukaryotic cells [23, 26] show that HA synthesis occurs at the reducing end. Purified SeHAS and SpHAS [27] or SpHAS in crude membranes [25] also add sugar-UDP units at the reducing end. The mechanism for polysaccharide biosynthesis is different if chain growth is from the reducing or nonreducing end. When a sugar is added from a sugar-nucleotide (making it the donor) to the nonreducing end of a polysaccharide (the acceptor), the nucleotide (e.g., UDP) is released. However, for reducing end elongation, the growing polymer chain is always attached to UDP. Reaction (1) shows the reaction for HA disaccharide assembly (D = disaccharide units). During HA synthesis, the UDP released at each transfer step comes from the HA-UDP intermediate formed by addition of the previous sugar. In each cycle of monosaccharide addition, the released UDP is derived from the last monosaccharide added:

(1) HAD-UDP+GlcUA-UDP ⟶UDP+HAD-GlcUA-UDPhhhhhhhhhh↓GlcNAc-UDPhhhhhUDP+HAD-GlcUA-GlcNAc-UDP

The donor HA-UDP transfers a hyaluronyl- (HA-) chain to the new sugar-UDP (acceptor) without cleavage of the latter high-energy linkage to UDP; the UDP released is from the HA-UDP donor. This situation is analogous to that for protein and fatty acid synthesis [48].

The IUBMB nomenclature for HAS glycosyltransferase activities (EC 2.4.1.212) is different compared to that for typical glycosyltransferases (Figure 1). An enzyme that utilizes GlcNAc-UDP to add to the nonreducing end of GlcUA would create a GlcNAc(*β*1,4)GlcUA linkage, whereas the HAS transferase activity adding GlcNAc-UDP at the reducing end creates the GlcUA(*β*1,3)GlcNAc linkage. Systematic naming of a transferase activity specifies the* donor: acceptor, group transferred*. Thus, addition of a GlcUA residue to a GlcNAc at the reducing end of the growing HA chain is catalyzed by an activity that adds a hyaluronyl chain from HA-GlcNAc-UDP to GlcUA-UDP. This is a HA-GlcNAc(*α*1→)UDP: GlcUA(*α*1→)UDP, *β*(1,4) hyaluronyltransferase. Similarly, an activity adding GlcNAc-UDP to a HA-GlcUA-UDP chain is a HA-GlcUA(*α*1→)UDP: GlcNAc(*α*1→)UDP, *β*(1,3) hyaluronyltransferase.

## 5. HA Translocation to the Cell Exterior Is Mediated by the HAS Protein Itself

The active sites of HAS and the sugar-UDP substrates are inside cells [28], so how do the large HA products (e.g., >40,000 sugars long; >8 MDa) reach the surface or extracellular space? Only the exogenous HAS protein (gene) and sufficient GlcNAc-UDP and GlcUA-UDP are required for HA biosynthesis and secretion by heterologous cells that cannot normally make HA, including* E. faecalis* [3],* B. subtilis *[38] and* D. melanogaster *[49]. Based on these findings and the lipid dependence and the topology of HAS, we proposed that HAS must have the ability to translocate the growing HA chain across the cell membrane into the extracellular space [11]. An alternative proposal was that an ABC transport system is required for the appearance of extracellular HA [50, 51], as for many bacterial polysaccharides [52]; HAS would synthesize intracellular HA that, while still being assembled, would be exported by a nearby membrane-bound ABC transport system.

It seemed unlikely that an ABC transport system was involved in HA translocation for several reasons: (i) It is unexpected that ABC polysaccharide transporters in* E. faecalis*,* B. subtilis,* and fruit flies would have such low specificity for their normal substrate that they would effectively transport HA. (ii) Since multiple MDs are not needed for just HA synthesis (e.g., the Class II* P. multocida* HAS contains one membrane anchor and, unlike Class I HASs, can be expressed as an active soluble truncated protein [53]), the topological organization of HAS enzymes, containing 6–8 membrane domains, is more consistent with a translocation function [54]. (iii) HAS activity is lipid-dependent or modulated by its lipid environment, consistent with an inherently intimate organization within the membrane bilayer, as expected for an HA translocation function, but not the independent ABC transport model. (iv) The sugar-UDP binding sites of Strep and mammalian HASs are at the inner membrane surface [46], which better fits a model in which the HA-UDP chain is extended near or within the membrane and translocated through the enzyme to the exterior (Figure 2). (v) Class I HASs are processive enzymes, meaning they do not release their HA-UDP chains during synthesis; dissociation of HA from HAS does not occur [5, 55]. This characteristic strongly supports a Pore model. HA-UDP that is bound by weak HA-HAS interactions would be released, moved, and elongated continuously within the enzyme, while still being retained by the topological constraint of being within a pore. In the ABC model, HA-HAS interactions are reversible, as for the nonprocessive PmHAS, which dissociates from and then rebinds HA after every sugar addition [53].

The strong biochemical logic supporting a Pore Translocation Model was confirmed by multiple studies showing that an ABC transporter Model for HA translocation is not correct. Thomas and Brown [56] found that ABC transporters are not involved in HA translocation by breast cancer cells and Medina et al. [57] showed that purified SeHAS mediates luminal dye efflux when added to liposomes, demonstrating the presence of an intraprotein pore. Hubbard et al. [58] found that SeHAS, incorporated into liposomes, delivers HA directly to the internal lumen, demonstrating that HAS possesses the predicted HA translocation function.

Misra et al. [59] showed that ABC transporter MDR1 expression is regulated by changes in the pericellular HA coat. Coordinately regulated expression of ABC transporters and HAS provides an alternative interpretation of studies implicating a role for transporters in HA transfer. Two independent cellular protective mechanisms (provided by pericellular HA coats and ABC multidrug transporters) may have coevolved in vertebrates to be coordinately regulated in a complex manner in response to environmental cues; this could explain why HAS and extracellular HA levels are lower in cells treated with inhibitors of multidrug transporter function [50]. Another explanation for inhibition of HA translocation by ABC transporter inhibitors is that these inhibitors alter uridine uptake or salvage pathways and change uridine nucleotide pools, which inhibits HAS (e.g., controls were not preformed to verify that substrate sugar-UDP levels did not decrease or that the potent HAS inhibitor UDP did not increase).

Finally, the finding that a bacterial cellulose synthase creates an intraprotein pore, in which the product cellulose is synthesized and translocated [60], confirms the principle we proposed in 1999 [11] that glycosyltransferases such as HA and cellulose synthases can mediate both polysaccharide synthesis and translocation.

## 6. HAS Synthesizes Chitin and Chitosyl-UDP Oligosaccharides

**Figure 3:**
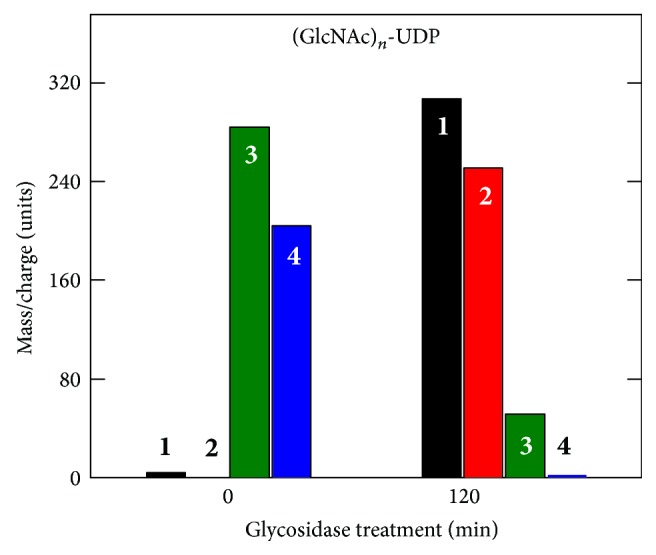
Glycosidase treatment converts larger (GlcNAc)_*n*_-UDP oligomers to GlcNAc-UDP. SeHAS membranes were incubated for 30 min with UDP-GlcNAc alone, Folch extracted, fractionated over a size exclusion column, and samples were either untreated (0 min) or treated (120 min) with jack bean hexosaminidase. The samples were then analyzed by MALDI-TOF MS to identify and quantify* m/z* signals of candidate oligomeric chitin-UDP fragments corresponding to *n* = 1–4 (boldface white or black numbers). The presence of chitin linkages was confirmed by the ability of glycosidase treatment to shift species with 3 or 4 sugars to products with 1 (GlcNAc-UDP) or 2 sugars. Additional MS/MS analysis of the starting sample ions (not shown) revealed smaller members of the expected oligomer series, including GlcNAc-UDP.

SeHAS synthesizes chitin oligomers, (GlcNAc-*β*1,4)_*n*_ [61], as reported for XlHAS1 [62] and MmHAS1 [63]. More importantly, however, and consistent with reducing end sugar addition, we found that SeHAS also makes novel chitin oligomers attached to -GlcNAc(*α*1→)UDP at the reducing end [61]. SeHAS incubated with only GlcNAc-UDP makes a series of (GlcNAc-*β*1,4)_*n*_-GlcNAc(*α*1→)UDP oligomers (for *n* = 2–15) corresponding to (GlcNAc)_2_-UDP through (GlcNAc)_7_-UDP products. SeHAS membranes incubated without substrate or with only GlcUA-UDP show no signals in this region. Product identifications were confirmed by MS-MS fragmentation and digestion with jack bean *β*-*N*-acetylglucosaminidase (e.g., all species ultimately yielded GlcNAc-UDP). For example, tri- and tetraoligomers were confirmed to contain *β*1,4-linked GlcNAc residues attached to GlcNAc-UDP because treatment with jack bean hexosaminidase converted almost all the initial oligomers to GlcNAc-UDP or (GlcNAc)_2_-UDP (Figure 3). Thus, HAS synthesizes (GlcNAc-*β*1,4)_1–7_-GlcNAc(*α*1→)UDP oligomers. These unusual sugar-nucleotide species, activated by *α*-attachment to UDP, are unstable and readily cleaved to yield chitin oligomers, explaining the ability of Class I HASs to make chitin.

**Figure 4:**
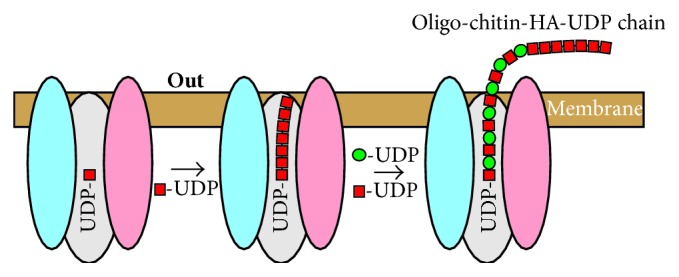
HAS initiation of HA synthesis using a self-made oligo-chitosyl-UDP primer results in HA chains with a chitin oligosaccharide cap at the nonreducing end. HAS makes chitin oligomers when incubated with GlcNAc-UDP (red squares) alone. We found that the enzyme first makes chitin oligomers linked to UDP at the reducing end [61], consistent with the mechanism of addition at the reducing end, and that these molecules could then serve as self-primers for HA disaccharide synthesis (GlcUA; green circles).

**Figure 5:**
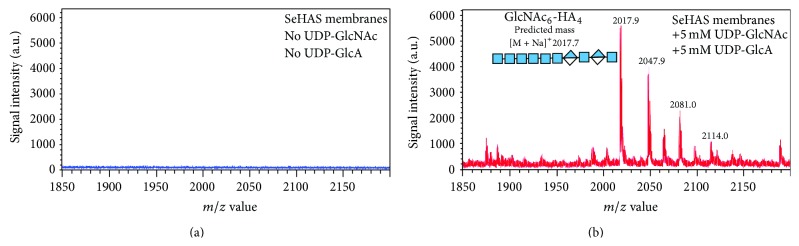
Mass spectral evidence for chitin-HA in HA made by SeHAS. Empty vector (a) or SeHAS (b) membranes were preincubated for 30 min with UDP-GlcNAc, UDP-GlcUA was added, and incubation proceeded for several minutes. The membranes were heated to release HA and subjected to two cycles of Folch extraction. Extracted HA products were speed vacuum concentrated, digested with ovine testicular hyaluronidase, and the resulting oligomers were subjected to affinity selection over carbograph. Eluted material was surveyed by MALDI-TOF MS to identify “hybrid” fragments corresponding to a chitin oligomer cap linked to HA disaccharides, such as the GlcNAc_6_-(GlcUA-GlcNAc)_2_ species shown.

Although HAS does not require an exogenous primer to make HA, these novel self-made (GlcNAc)_*n*_-UDP products could potentially serve as endogenous primers that enable HAS to initiate HA chain assembly. In this proposed scenario (Figure 4), the nonreducing end of all HA chains retains this initial chitin oligomer primer, and all HA molecules have a novel non-HA structure (a chitin oligosaccharide cap) at their nonreducing end. Ongoing studies support this hypothesis [64], including HAS-dependent* m/z* signals indicating hybrid chitin-HA species such as GlcNAc_6_(GlcUA-GlcNAc)_2_ in ovine testicular hyaluronidase-digested samples (Figure 5(b)). Empty vector membranes without HAS (Figure 5(a)) or SeHAS membranes incubated without substrate (not shown) show no signals in this region. The hybrid chitin-HA digestion fragments can be affinity purified, fractionated by TLC or PAGE, and shown to contain multiple nonreducing GlcNAc residues that are releasable by treatment with jack bean hexosaminidase, as in Figure 3. Since we studied chitin-UDP oligomer products* in vitro* only, under conditions (e.g., exposure to a single sugar-UDP) that may not normally be encountered in cells, it remains to be demonstrated if these interesting products are also made* in vivo*. Studies are in progress to determine if HA molecules made by streptococcal and mammalian Class I HASs contain a nonreducing end chitin oligosaccharide cap. The presence of a chitin cap would have important physiologic implications for the polarity of HA chains and the potential ability of chitin-like binding proteins in the biomatrix or on cell surfaces to orient, align, and organize individual HA polymers into bundles; for example, cables or fibers [65].

## 7. Class I HAS Performs Multiple Binding and Catalytic Function in Order to Synthesize HA

We noted above the seven functions that all Class I HAS enzymes possess in order to catalyze the steady-state assembly of GlcNAC(*β*1,3)GlcUA(*β*1,4) disaccharides during HA synthesis. The recent discovery that HASs are also able to make oligo-chitosyl-UDP species and that these can serve as self-primers for subsequent HA assembly means that this enzyme family also possesses more binding site and catalysis functions than previously recognized. These functions are summarized in Box 1, using the standard transferase nomenclature, and listed in order of use in the initiation and assembly of HA. The synthesis of chitin-UDP species requires three binding sites with different structural specificities and two transferase activities (Box 1(A), functions 1–5). Initiation of HA synthesis requires making the first disaccharide using a non-HA substrate, so an acceptor site for the GlcUA to be added and a unique transferase activity (used only once for each HA molecule synthesized) are needed (Box 1(B), functions 6 and 7). Subsequent steady-state HA disaccharide synthesis requires seven functions, using two binding sites noted in (A) and (B) (functions 2 and 6), two additional hyaluronyl-UDP species binding sites, and two additional corresponding transferase activities (Box 1(C), functions 8–11). The final function is the translocation activity of HAS, which acts in a continuous manner during HA chain elongation but is listed separately to emphasize its novel and separate nature, as a “spatial” rather than chemical catalytic process (Box 1(D), function 12). Thus, an astounding 12 discrete functions are attributable to Class I HASs in order for these enzymes to initiate, assemble, and transfer the oligo-chitosyl-HA-UDP polysaccharide to the cell exterior; it is not known if released HA chains are still attached to UDP at their reducing ends or if chains are released because this group has been lost and elongation has therefore stopped, resulting in HA release.

## 8. A Pendulum Model for Polysaccharide Translocation

**Figure 6:**
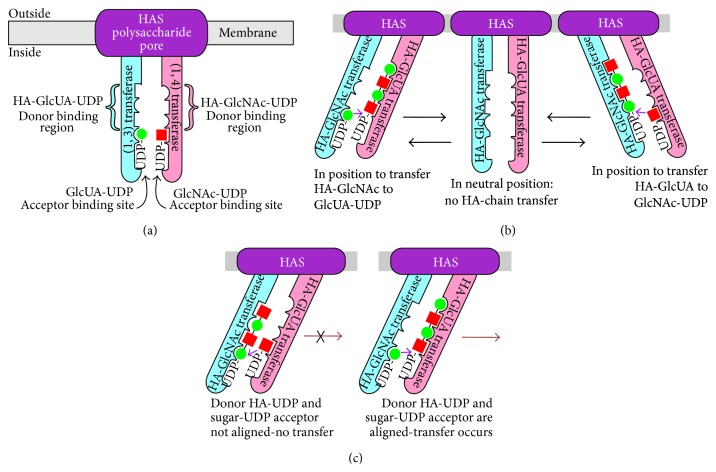
The Pendulum Model for HAS Translocation of HA. (a) Organization of hyaluronyl transferase domains and glycosyl-UDP binding sites within HAS. The scheme shows the cell membrane (gray) and three overall domains of the HAS·lipid complex: a pore region (purple) containing HAS MDs through which a growing HA chain is passed to the exterior, and two catalytic domains that behave as swinging arms (blue and pink). Each arm contains one of the two functional hyaluronyl transferase activities and binding sites needed to add either an HA-GlcUA-UDP donor chain to GlcNAc-UDP [left arm (blue); the *β*(1,3)-hyaluronyl transferase] or an HA-GlcNAc-UDP donor chain to GlcUA-UDP [right arm (pink); the *β*(1,4)-hyaluronyl transferase]. The figure also illustrates that an individual sugar-UDP binding site is part of the HA-UDP binding site on each arm. (b) The interactions between the glycosyl-UDP binding sites and hyaluronyl transferase domains change as the domain arms move. The three positions, from left to right, indicate three conformations in which HAS is either able to create the GlcNAc*β*(1,4)GlcUA bond, unable to perform either transferase function, or able to create the GlcUA*β*(1,3)GlcNAc bond. The left and right positions also illustrate that when the enzyme is in position to catalyze one of the transferase reactions, the growing HA chain is bound primarily to one arm, whereas the sugar-UDP substrate is bound to the other arm. In the central panel, a neutral or inactive position, the individual sugar binding sites in the HA-binding region on each arm are “misaligned” so that they are unable to bind HA at the same time. (c) HAS hyaluronyl transferase activities require correct alignment between the glycosyl-UDP binding sites on opposite domain arms. Transferase function depends on how the two arms are aligned with respect to the ability to bind substrates or to perform catalysis. The relative positioning of HA-UDP and sugar-UDP binding sites are shown with the complementary glycosyl-UDP substrates bound incorrectly and not aligned for creating a glycoside bond (left) or with the right substrates bound and aligned correctly for successful -GlcNAc-*β*(1,4)-GlcUA-bond formation (right).

**Figure 7:**
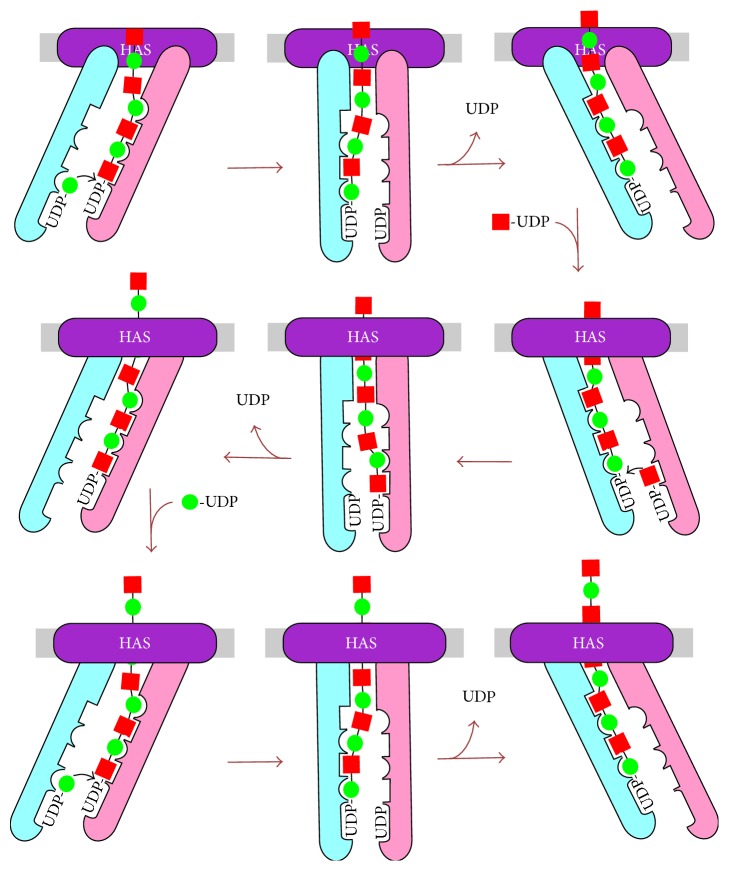
The Pendulum Model: Arm movement and HA transfer between arms drives HA chain translocation through the HAS·lipid complex to the cell exterior. The changing alignment of the HA-binding regions on the two arms in the two extreme positions (left and right) creates the ability of the enzyme to move the HA chain from one arm to the other (shown in each row). When the arms swing from one extreme position to the other, the HA chain is transferred from the first arm to the other arm as the HA-binding site alignments move out of (neutral position) and then back into register. A “time-lapse” of HAS action is illustrated in the nine panels as the enzyme adds three new sugars to an HA-UDP chain of seven sugars. The enzyme goes through three stages of arm movement (in each row) to add each new sugar. After assembly of each disaccharide, the enzyme arms are in the same starting position (e.g., the left panels in top and bottom rows). The sugars “crossing” the membrane are shown outside of the enzyme in the top row to help orient the reader, and then within the intraprotein pore in the middle and bottom rows. An animation of this process showing chain translocation through assembly of an HA 10-mer is at http://www.glycoforum.gr.jp/science/hyaluronan/HA06a/Pendulum_Hypothesis_Anima.files/slide0001.htm.

We proposed a novel mechanism in 2004 [37] by which a single membrane-bound HAS·lipid complex could simultaneously extend a polymer chain at its reducing end and extrude the growing chain through the membrane (Figures 6 and 7), in a process not requiring other proteins or ATP. The model also applies to other membrane polysaccharide synthases that use two transferase sites to make hetero- or homopolysaccharides (e.g., cellulose). The model involves continuous “swinging” movement by enzyme domains (pendulum-like) and has variations (three of which are noted in Table 1), depending on whether the catalytic mechanism utilizes independent glycosyl-UDP binding sites (e.g., variants 1 and 2) or one site with alternating specificity (e.g., variant 3). Disaccharide assembly in variant 1 or 2 is sequential or simultaneous, respectively, whereas assembly would necessarily be one sugar at a time in a variant 3 mechanism. Key features of the Pendulum Model are presented below to describe Pendulum Model variant 1, but similar central points and considerations apply to variant 2.

*(i) HAS Has Two Functional Domains That Act as “Arms.”* Each arm contains an active site for one of the glycosyltransferase functions, a binding site for one of the acceptor sugar-UDPs, and a binding site for one of the donor HA-UDP species (Figure 6(a)). The right arm (pink) contains the GlcNAc-UDP acceptor binding site, the HA-GlcNAc-UDP donor binding site, and the (1,4) hyaluronyltransferase activity that makes the GlcNAc-*β*(1,4)-GlcUA linkage. Some residues participating in interactions (e.g., binding) needed for each function might be in either arm or in other HAS domains not shown in Figures 6 and 7.

*(ii) Only One Arm Is Active at a Time and Their Activities Are Reciprocal*. When one arm is active as a transferase, the other serves as an acceptor binding site. Each arm can “swing” to one of three functionally different positions (Figure 6(b)) that correspond to conformations in which it is active as a donor binding site and transferase, inactive, and active as an acceptor binding site. For example, the right (pink) arm can be active as a donor binding site for HA-GlcNAc-UDP and the (*β*1,4) transferase (Figure 6(b), left), inactive (Figure 6(b), center), or active as an acceptor binding site for GlcNAc-UDP (Figure 6(b), right). In the same relative positions, the left arm is similarly active as an acceptor binding site for GlcUA-UDP (Figure 6(b), left), inactive (Figure 6(b), center), or active as the (*β*1,3) transferase and donor binding site for HA-GlcUA-UDP (Figure 6(b), right). The relationship of the arms in a neutral inactive conformation (Figure 6(b), center) creates misalignment of glycosyl-UDP acceptor and donor sites that is not suitable for glycosyl binding or transfer. In contrast, when the arms have moved to either the left or the right position, the alignment of glycosyl-UDP donor and acceptor are favorable for the respective transferase activity to function. In either of the two functional positions, in which transferase activities add sugar-UDP to the growing HA-UDP chain, there is only one appropriate active arrangement of all the binding and catalytic sites (Figure 6(c)). Even if binding of the alternate acceptor and donor could occur, the active sites would not be aligned for functional sugar transfer (Figure 6(c), left).

*(iii) HA-UDP Translocation Is Coupled to Sugar Addition*. As each arm moves from one side to the other through a sugar addition cycle, the HA chain is extruded through the enzyme and lipid bilayer (each row in Figure 7) one sugar at a time (or two at a time if concerted disaccharide synthesis occurs). With each new sugar-UDP addition, the bound HA-UDP chain is passed from one HAS arm to the other (e.g., like a person pulling up a rope, hand-over-hand) and as the arms swing, the nonreducing end of bound HA-UDP is simultaneously moved away from the intracellular active sites. The synchronized arm movement provides the force needed to move the bound HA-chain through the protein pore and cell membrane to the cell exterior. The bound HA-chain does not dissociate from HAS during continuous elongation because it is always bound to one arm or the other as it cycles between the two arms and it is always topologically constrained by being within the HAS-lipid pore.

*(iv) Other Variants of the Pendulum Model*. The general scheme for other variants of the Pendulum Model is slightly different compared to that for variant 1 (Table 1). In variant 2 the three glycosyl-UDP binding sites might be in one arm, but the transferase sites and the HA-binding sites for moving the chain could be on the other arm or both arms. The glycosyl-UDP binding sites could be on two arms in variant 3, and simultaneous disaccharide assembly could occur in swinging from one position to the other. Release of UDP products could occur as the arms swing to the other position, transfer (and translocate) the HA-UDP chain, and “reload” for another round of disaccharide assembly. An alternating specificity mechanism (variant 3) involving fewer sites, whose glycosyl-UDP binding specificity cycles between two species (e.g., HA-GlcNAc-UDP and HA-GlcUA-UDP), is intrinsically more complicated and may thus be less likely, especially given the large number of potential glycosyl-UDP binding sites in the Class I HAS family (Figure 2 and Section 10 below).

A key mechanistic feature to consider for concerted addition of a disaccharide unit to HA-UDP by adding both UDP-sugars simultaneously is that HAS would use only one of the two possible HA-UDP donors, either HA-GlcUA-UDP or HA-GlcNAc-UDP and thus make only one of the two possible HA disaccharide units (i.e., HA-GlcUA(*β*1,3)GlcNAc-UDP versus HA-GlcNAc(*β*1,4)GlcUA-UDP, resp.). Although no data currently support one mechanism over another, the presence of a chitin cap at the nonreducing end defines the first HA disaccharide made (Box 1) as GlcNAc(*β*1,4)GlcUA and makes it likely that subsequent coordinated disaccharide assembly would utilize the three bound substrates indicated in reaction (2), so that donor HA-GlcUA-UDP will be covalently linked to GlcNAc-UDP, as it in turn is linked to GlcUA-UDP in sequential coupled reactions that would be essentially simultaneous:

(2) HA-GlcUA-UDP+GlcNAc-UDP+GlcUA-UDP   ⟶HA-GlcUA-GlcNAc-GlcUA-UDP+2UDP

## 9. The Bioenergetics of HA Translocation

Any mechanism of HA translocation by HAS must account for several potential bioenergetic obstacles: (i) the energy barrier associated with transfer of a hydrophilic HA chain across a hydrophobic membrane lipid bilayer, (ii) the energy required to move an HA molecule might become progressively greater as it elongates to larger mass and hydrodynamic volume [66], and (iii) the energy to cause conformational changes and movement of domains within the enzyme that could be physically responsible for rapid chain movement across the membrane without releasing the chain. For perspective, an 8 MDa HA chain proportionally enlarged to human scale would be equivalent to a 1 mm wide thread >30 m long.

*(i) How Does HAS Overcome the Hydrophobic Membrane Barrier?* Membrane transporters, such as lac permease with 12 MDs [67], mediate sugar transfer through an intraprotein pore created by interactions among many MDs. HASs appear to compensate for not having enough MDs to form such an intraprotein pore by their interactions with phospholipids [11, 32]. HAS·lipid interactions could allow the enzyme to create a larger pore-like passage within the enzyme, through which the growing HA chain could pass [11, 57, 68]. HAS·lipid complexes would present exterior hydrophobic interactions with fatty acyl groups in the bilayer, while engaging HA on the interior pore surface with hydrophilic interactions; thus, an HA chain moving through a HAS·lipid pore could effectively bypass the hydrophobic membrane barrier.

*(ii) How Could HAS Move HA Chains with Masses That Get Progressively Greater?* Intuitively, one might expect that the energy needed to overcome the inertia of, and move, a long HA-UDP chain (e.g., 4 MDa) would be much greater than the energy needed to move a short HA oligosaccharyl-UDP chain (e.g., 4 kDa). However, two factors may make this apparent “molecular weight-lifting” less difficult than it appears.

- *HA Segmentation*. The segmented, semi-independent movement of discrete portions (roughly 100 sugars long) of a large HA chain [69–71] could create an upper limit for the “apparent” mass of HA that HAS would need to “move” to translocate a discrete section. If segments of ~100 sugars behave semi-independently for brief periods (e.g., msec), during which translocation is favored, then the apparent mass of HA against which HAS needs to generate a translocation force would only be ~20 kDa. As a chain was initiated and elongated, HAS might initially translocate it rapidly and then progressively more slowly until the segmental length was approached, and a steady-state translocation rate was then reached. The observed kinetic profile for how HA mass increases during synthesis begins very rapidly and then becomes slower [55].
- *Increased Release Forces*. The net forces generated by Brownian motion may tend to pull the HA chain away from the enzyme and thus facilitate the translocation action of HAS. A possible chain release mechanism, in fact, could be the balance between the Brownian and other forces acting to release the HA and the HA-binding forces mediated by sites within the enzyme acting to retain the HA [66]. In contrast, cells with a pericellular HA coat might effectively stabilize and stop HA synthesis, because the presence of nearby HAS-HA complexes could augment and increase the forces favoring HA retention. As HA chain density increased at the cell surface, the network of interacting chains would diminish the effects of Brownian-generated forces acting to release the HA, thus resulting in a stabilized gel-like cell surface coat still anchored to multiple HAS molecules. In the extreme of this scenario, HA synthesis would slow or stop as forces generated by the HA coat network increased, finally providing too much resistance for HAS to translocate HA.

**Figure 8:**
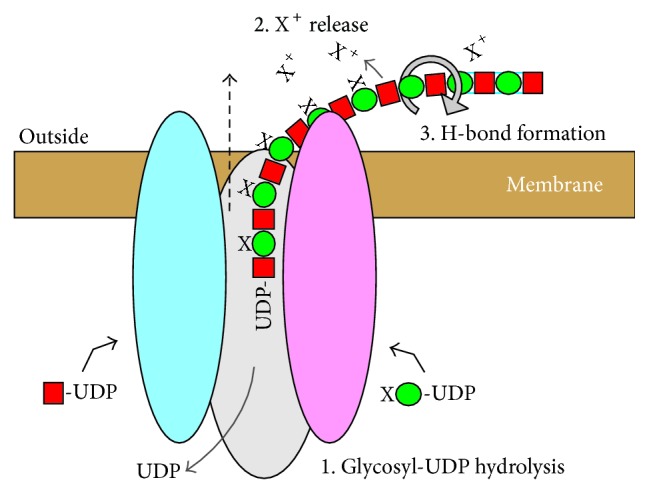
Three energy sources may drive the HAS-mediated translocation of HA-UDP. The diagram illustrates three sources of energy (numbers 1–3) that could contribute to an overall favorable free energy change to drive translocation (dashed black arrow), equivalent to ~1 ATP per disaccharide, as discussed in the text. 1. Two HA-UDP bonds are hydrolyzed to make two glycoside bonds per disaccharide. 2. Energy can be captured by extracellular release of a cation (X^+^; bound to the intracellular GlcUA-UDP substrate and incorporated into HA by HAS) as the GlcUA is released from HAS and any associated restraints on the –CO_2_X group by the translocation process. Subsequent reactions in the extracellular environment (e.g., ion-pair association, dissociation, or exchange) and the coupling of a released ion, such as K^+^, to a cellular electrochemical gradient (potential) would provide favorable energetics. 3. Up to four H-bonds (blue lines between sugars at far right) could be formed as each new GlcNAc-GlcUA disaccharide (red squares and green circles, resp.) is released to the exterior, free of constraints imposed by being bound to HAS. For example, two H-bonds between the released disaccharide GlcNAc and GlcUA and two H-bonds between the GlcUA in the disaccharide released during the previous synthetic cycle and the new disaccharide GlcNAc. The gray circular arrow indicates a glycosidic bond that rotates (e.g., so that the N-acetyl and carboxyl group of adjacent sugars are on the same side of the chain) allowing the formation of new H-bonds.

*(iii) What Are the Energy Sources for HA Translocation?* At least three possible energy sources could contribute to an HA translocation mechanism (Figure 8), as estimated below: glycosyl-UDP hydrolysis [4–8 kJ/mol], H-bond formation [12–24 kJ/mol], and ion-pair reactions and the potential energy of electrochemical gradients [4–8 kJ/mol]. If these energy sources contribute to the HA translocation mechanism, a conservative estimate of the total free energy change for HA translocation is ~30 kJ/mol or ~7 kcal/mol (1 kcal = 4.18 kJ) of HA disaccharide units moved across the membrane. This is equivalent to 1 ATP and would be a favorable bioenergetic situation, explaining how an ATP-independent translocation process is feasible.

(a) *UDP-Sugar Hydrolysis*. The free energy for Glc-UDP hydrolysis is 7.3–7.6 kcal/mol and the difference between Glc-UDP hydrolysis and glucoside bond formation is ~0.6–1.0 kcal/mol [72]. Values for hydrolysis of GlcNAc-UDP and GlcUA-UDP are not available but are presumably somewhat greater, due to destabilizing repulsive and steric factors. Free energy differences between sugar-UDP hydrolysis and glycoside bond formation for GlcNAc and GlcUA are likely similar to Glc, ~0.6–1.0 kcal/mol. Thus, the available “excess” free energy to perform additional work beyond creating two glycoside bonds could be ~1-2 kcal/mol (~4–8 kJ/mol) of HA disaccharide units (Figure 8, #1).
(b) *Electrochemical Gradients or External Ion Reactions May Provide Additional Energy*. HA translocation to the cell exterior must necessarily be associated with one or more electrochemical gradients that could provide additional energy for the translocation process. HAS might recognize only one of ≥4 possible UDP-GlcUA species as the correct substrate during catalysis, depending on the state of the carboxyl group. The carboxyl group could be dissociated and free (i.e., an anion) or neutral and bound to either H^+^, Na^+^, or K^+^ (Mg^+2^ is also a possibility). In most of these cases, a favorable energetic situation would occur when newly added GlcUA is transferred to the exterior: protonated carboxyl groups would readily dissociate releasing H^+^, negatively charged carboxyl groups would bind to extracellular cations (e.g., Na^+^), and bound K^+^ ions would exchange with Na^+^ ions, which are in excess. Such ion-pair reactions would yield energy (that is difficult to estimate) and tend to increase (pull) the equilibrium for translocation, in a manner similar to removal of a reaction product.
 The situation with bound K^+^ might be particularly favorable because HA translocation would also then be coupled to the cellular K^+^ ion gradient (high intracellular and low outside), providing an additional energy source for overall HA synthesis and translocation [73]. Given the intracellular abundance of K^+^, ion composition and pH, the K^+^-GlcUA-UDP species is likely the most abundant form of GlcUA-UDP and, thus, likely to be the normal HAS substrate. Consistent with this, purified SeHAS is more active in the presence of K^+^ than Na^+^ ions [33]. The energy available if one K^+^ per disaccharide is transferred to the extracellular environment is ~1-2 kcal/mol (4–8 kJ/mol) of HA disaccharides (Figure 8, #2). This value, as noted above, is likely underestimated because it lacks the contributions from associated ion-pair reactions.
(c) *H-Bond Formation*. Experimental and modeling studies [69, 71, 74, 75] indicate that HA forms multiple intrachain H-bonds, by creating up to two H-bonds between adjacent sugars (similar to H-bonds between amino acids to form protein *α*-helices). Additional energy could be captured for chain translocation if HAS couples the formation of several new H-bonds with HA chain movement and the rotation of alternate newly added sugars occurring within or just external to the HAS pore (Figure 8, #3). Assuming an average value of ~6 kJ/mol (~1.5 kcal/mol) for intra-HA H-bonds, then translocation could yield up to 12–24 kJ/mol (3–6 kcal/mol) associated with the formation of 2–4 H-bonds per mol HA disaccharides.

## 10. Class I HASs Contain Multiple Potential Glycosyl-UDP Binding Sites

HASs contain only one conserved “DXD” motif (DSD^161^ in SeHAS; Figure 2), typically found in the active sites of many glycosyltransferases and whose carboxyl groups are coordinated with Mg^+2^ and the phosphoryl groups of a nucleotide-sugar [76]. However, the same “DXD” function can be mediated by essentially any cluster in which two of three contiguous residues are Asp or Glu [77]. StrepHASs also contain a second DAD^153^ motif, which is conserved in the HAS family, except for chicken HAS2 and chlorella HAS (Asp^153^ is conserved in all HASs). A DAE^79^ motif in SeHAS and SpHAS, but not in SuHAS, is present in 10 of 13 eukaryotic HASs. Related “XDD” motifs are also present in all HASs at multiple positions: for example, SeHAS GDD^260^; ED^77^ (EN in* Chlorella* HAS); ED^116^ (conserved positionally as EE, EXE, DE, or EXD). Five of the seven “XDD” motifs conserved in the StrepHASs are conserved positionally (i.e., within ≤6 residues) in all HASs, with the exception of XlHAS1 (in which 3 of the 5 motifs are conserved, but five others are also present). Thus, all Class I HASs except XlHAS1 contain at least six conserved DXD or XDD motifs and, therefore, all HASs potentially have enough glycosyl-UDP binding sites to assemble a disaccharide unit in either a coordinated or simultaneous manner (Table 1).

## 11. HAS as a Glycosyltransferase Family Member and Consequent Structural Predictions

HAS is a member of the GT2 family [78–80] in the CAZy database (http://www.cazy.org/) and catalyzes an inverted mechanism (i.e., creating a *β*-linked glycoside from the *α*-linked precursor). These family members require a divalent metal ion and contain at least one DXD motif and a GT-A fold (related to the Rossmann fold), consisting of a seven-stranded *β*-sheet flanked by *α*-helices. The active site in GT-2 proteins is created by the association of the large *β*-sheet with a smaller *β*-sheet. Present in perhaps >5,000 proteins, a basic GT-A fold provides a versatile “platform” for creating a variety of catalytic situations.

HAS is an exceptional GT2 family member due to its multiple MDs, which might create inherent topologic constraints in regions of homology to the GT-A fold (making modeling essentially impossible). Based on extensive sequence similarity through a ~260 aa region between MD2 and MD4 (Figure 2), the HAS catalytic domain might adopt an overall fold similar to other GT2 family members, including cellulose synthase [81]. Similarities between HA and cellulose synthases include N-terminal nucleotide binding motifs, a putative catalytic base, a QxxRW motif associated with processivity, and that both proteins create an intraprotein pore for their products [58, 60]. The GT2 family model predicts that the N-terminal subdomain binds the sugar-UDP donor and the C-terminal subdomain binds the acceptor. For enzymes such as HAS that add to the reducing end the normal designation of donor and acceptor binding sites may be switched. Thus, in HAS an N-terminal subdomain binding site for sugar-UDP could be an acceptor site. Alternatively, if this was conserved as a donor site, then it would accommodate an HA-UDP substrate. The ultimate answers to many of the molecular and mechanistic questions posed here will likely only be revealed when the structure of HAS is ultimately determined at sufficient atomic resolution.

## 12. Conclusions

Since the first HAS gene was cloned in 1993, the field has progressed and answered important questions about the molecular basis of HA synthesis. In particular, there is a growing consensus that membrane-bound Class I HAS enzymes are lipid-dependent and active as both protein monomers or HAS multimers associated with specific lipids (and cholesterol) and that the Strep and mammalian HASs assemble HA at the reducing end. It is now resolved that HAS uses intracellular precursors and couples HA synthesis with HA translocation to the cell surface or biomatrix. Elucidation of this multifunctional process will surely reveal more surprises and unexpected intricacies about the complicated orchestration of molecular processes employed by a single enzyme protein to assemble and simultaneously translocate one of the largest single biomolecules made in the animal or microbial kingdoms. Future structural studies will reveal whether the general principles of the Pendulum Model are able to explain the mechanism of how HAS couples HA synthesis with translocation. Understanding how these enzymes work to synthesize and translocate HA will likely provide opportunities to identify therapeutics and to better understand how HASs are involved in normal human health and in tumorigenesis, metastasis, and inflammatory diseases such as arthritis.

## Figures and Tables

**Box 1:**
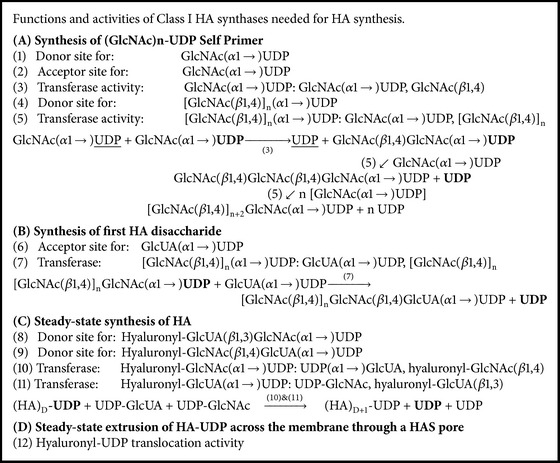
Summary of HAS functions required for HA biosynthesis. At least twelve discrete binding and catalytic function are required for Class I HA synthases to create a (GlcNAc)_*n*_-UDP self-primer (A, functions 1–5), to initiate HA disaccharide synthesis (B, functions 6 and 7), and to then assemble HA disaccharide units in a continuous manner (C, functions 8–11), while the growing HA-UDP chain is also continuously translocated through the HAS·lipid complex pore to the cell surface or exterior (D, function 12). Two functions for steady-state HA disaccharide synthesis (C) are also used to make (GlcNAc)_*n*_-UDP (#2) or the first HA disaccharide (#6).

**Table 1:** Three variations of the Pendulum hypothesis.

Variant	Disaccharide assembly	Glycosyl-UDP sites
1	Sequential	Four independent sites
2	Simultaneous	Three independent sites
3	Alternating	Two or three dependent sites

The mechanism for adding sugars to the reducing end of HA could entail polymerization of a disaccharide unit by either a sequential (i.e., one sugar at a time) or a concerted (i.e., simultaneous) mechanism. For the sequential assembly of a disaccharide unit (variant 1), the enzyme would need two glycosyl-UDP binding sites for addition of each sugar, one for a HA-UDP and one for a sugar-UDP. Since there are two types of HA-UDP species, the enzyme would need four glycosyl-UDP binding sites to assemble each disaccharide unit. For disaccharide assembly at the reducing end in a concerted way (variant 2), HAS would require three glycosyl-UDP binding sites, one each for GlcNAc-UDP, GlcUA-UDP, and a specific HA-UDP donor chain (i.e., HA-GlcUA-UDP or HA-GlcNAc-UDP), depending on which of the two possible HA disaccharide units was assembled. Another variation is that HAS contains only one donor and one acceptor glycosyl-UDP binding site, whose specificities alternate as the two sugars are assembled one at a time (variant 3). If there is a single donor binding site, its specificity would alternately recognize HA-GlcUA-UDP and HA-GlcNAc-UDP. There could be two separate sugar-UDP sites, but if there is a single acceptor binding site, its specificity would also alternate in a reciprocal fashion with the HA-UDP site to bind GlcUA-UDP or GlcNAc-UDP.

## Abbreviations

CL: Cardiolipin
GlcNAc-UDP: The nucleotide-sugar with the reducing end at the right
GlcUA-UDP: The nucleotide-sugar with the reducing end at the right
HA: Hyaluronic acid, hyaluronate, and hyaluronan
HA-: A hyaluronyl group
HA-UDP: A hyaluronyl group with *α*-linked UDP at the reducing end
HAS: HA synthase
MD: Membrane domain
SeHAS: *Streptococcus equisimilis* HAS
SpHAS: *Streptococcus pyogenes* HAS
StrepHAS: Streptococcal HA synthases
SuHAS: *Streptococcus uberis* HAS
XlHAS: *Xenopus laevis*.
